# Gender-specific mortality in DTP-IPV- and MMR ± MenC-eligible age groups to determine possible sex-differential effects of vaccination: an observational study

**DOI:** 10.1186/s12879-015-0898-8

**Published:** 2015-03-24

**Authors:** Tessa M Schurink-van’t Klooster, Mirjam J Knol, Hester E de Melker, Marianne AB van der Sande

**Affiliations:** Centre of Epidemiology and Surveillance of Infectious Diseases, Centre for Infectious Disease Control, Bilthoven, the Netherlands; Julius Centre for Health Sciences and Primary Health Care, University Medical Centre Utrecht, Utrecht, the Netherlands

**Keywords:** Vaccination, DTP-IPV, MMR, MenC, Mortality, Sex-differential

## Abstract

**Background:**

Several studies suggested that vaccines could have non-specific effects on mortality depending on the type of vaccine. Non-specific effects seem to be different in boys and girls. In this study we want to investigate whether there are differences in gender-specific mortality among Dutch children according to the last vaccination received. We tested the hypothesis that the mortality rate ratio for girls versus boys is more favourable for girls following MMR ± MenC vaccination (from 14 months of age) compared with the ratio following DTP-IPV vaccination (2–13 months of age). Secondarily, we investigated whether there were gender-specific changes in mortality following booster vaccination at 4 years of age.

**Methods:**

This observational study included all Dutch children aged 0–11 years from 2000 until 2011. Age groups were classified according to the last vaccination offered. The mortality rates for all natural causes of death were calculated by gender and age group. Incidence rate ratios (IRRs) were computed using a multivariable Poisson analysis to compare mortality in boys and girls across different age groups.

**Results:**

The study population consisted of 6,261,472 children. During the study period, 14,038 children (0.22%) died, 91% of which were attributed to a known natural cause of death. The mortality rate for natural causes was higher among boys than girls in all age groups. Adjusted IRRs for girls compared with boys ranged between 0.81 (95% CI 0.74-0.89) and 0.91 (95% CI 0.77-1.07) over the age groups. The IRR did not significantly differ between all vaccine-related age groups (p = 0.723), between children 2–13 months (following DTP-IPV vaccination) and 14 months - 3 years (following MMR ± MenC vaccination) (p = 0.493) and between children 14 months - 3 years and 4–8 years old (following DTP-IPV vaccination) (p = 0.868).

**Conclusions:**

In the Netherlands, a high income country, no differences in gender-specific mortality related to the type of last vaccination received were observed in DTP-IPV- and MMR ± MenC eligible age groups. The inability to detect this effect indicates that when non-specific effects were present the effects were not reflected in changes in the differences in mortality between boys and girls. The findings in this large population-based study are reassuring for the continued trust in the safety of the national vaccination programme.

## Background

The Dutch National Immunisation Programme (NIP) has contributed greatly to reduced mortality among infants and (young) children. Since its introduction in 1957, the number of deaths due to diseases targeted by the NIP has drastically declined [1]. Notwithstanding this important contribution of vaccinations, several studies in recent decades have suggested that vaccinations could also give non-specific effects, i.e. effects on non-targeted diseases, on mortality, with differential effects in boys and girls [2-17]. It is therefore of great public health relevance to explore the overall impact of vaccination on mortality.

Studies in countries with high childhood mortality observed that live attenuated vaccines, such as the measles vaccine and BCG-vaccine, might reduce mortality far more than can be attributed to the target disease [2,3,7,8,10,12-16]. In contrast, it has been suggested that inactivated vaccines, such as DTP, can increase the mortality from infections other than the target disease [2-8,10,11,13-17]. Such non-specific effects can be significant, with changes in all-cause mortality of more than fifty percent. Both positive and negative effects were stronger in girls than in boys [2,3,5,6,9,10,13-17]. Several mechanisms driving potential sex-differential effects of vaccines have been suggested, including differences in adaptive and innate immunity [2,18].

In the Netherlands, no research has been performed to date on possible non-specific effects of vaccination on mortality. However, such potential effects of vaccinations need to be explored proactively for public health purposes to guide vaccination policy, including ensuring accurate information to the public and maintaining public trust in public health interventions such as vaccinations. To assess whether there are such non-specific effects of vaccines, mortality in vaccinated and unvaccinated children should be compared, ideally in a randomised controlled trial. However, the use of randomised controlled trials is not feasible or ethical in this case, as the direct benefits of vaccination have been demonstrated very convincingly. In the Netherlands, vaccination coverage is high, and childhood mortality is low. Therefore, in this study, we retrospectively investigated whether there were indications for differences in gender-specific mortality among children with respect to the ages of last vaccination offered. We hypothesized that the mortality rate ratio for girls versus boys is more favourable for girls following MMR ± MenC vaccination (after 14 months of age) compared with the ratio after DTP-IPV vaccination (2–14 months of age). Secondary we investigated whether there are gender-specific changes in mortality following booster vaccination at 4 years of age.

## Methods

### Study design and population

For this observational study, a dynamic cohort was used that included all Dutch children who were 0-11-years-old in the years 2000 to 2011. The date of study entry was 01/01/2000 or the date of birth, whatever was last. The end date of follow up was 31/12/2011, the date of 12th birthday or the date of death, whatever came first. The data were available from Statistics Netherlands, who linked demographic data of persons included in the Municipal Personal Records Database (GBA) with causes of death derived from Statistics Netherlands.

### Dutch national immunisation programme

The Dutch NIP was started in 1957 with childhood vaccinations against diphtheria, tetanus, pertussis and poliomyelitis. The programme has been regularly updated and modified ever since its inception. The current programme contains childhood vaccinations against diphtheria, tetanus, pertussis, polio, *Haemophilus influenzae* serotype b, mumps, measles, rubella, meningococcal serogroup C (MenC), hepatitis B, pneumococcal disease and human papillomavirus (Table 1). Table 2 presents the main changes in the childhood NIP programme in the period 1988 to 2011. Some of the important changes are the introduction of meningococcal C vaccination in 2002 at 14 months of age (i.e., administered together with the MMR vaccine) with in addition a catch-up campaign up to 19 years of age, the expansion of the DTP-IPV vaccine with *Haemophilus infuenzae* type b and hepatitis b components in 2003 and 2011, respectively, and the introduction of pneumococcal vaccination in 2006 at 2, 3, 4 and 11 months of age (simultaneous with DTP-IPV/Hib).

**Table 1:** **Vaccination scheme and coverage of the Dutch NIP in 2011**

**Age**	**Vaccination 1**	**Coverage vaccine 1 (%)**	**Vaccination 2**	**Coverage vaccine 2 (%)**
2 months	DTaP-HBV-IPV/Hib		Pneumo	
3 months	DTaP-HBV-IPV/Hib		Pneumo	
4 months	DTaP-HBV-IPV/Hib		Pneumo	
11 months	DTaP-HBV-IPV/Hib	95.4/96.0	Pneumo	94.8
14 months	MMR	95.9	MenC	95.9
4 years	DTaP-IPV	92.0		
9 years	DT-IPV	92.2	MMR	92.1
12 years	HPV (3 doses)*	52.5		

*Only for girls; three doses on 0 days, 1 month and 6 months.

**Table 2:** **Major changes in the Dutch NIP from 1988 to 2011**

**Month/Year**	**Change**
July 1993	Introduction of vaccination against *Haemophilus influenzae* type b (Hib) disease for all children born on or after 1^st^ April 1993
January 1999	Advanced age for start of primary vaccinations from 3 to 2 months
July 2001	Acellular pertussis vaccine added at 4 years of age for all children born on or after 1^st^ January 1998
September 2002	Meningococcal serogroup C vaccine added at 14 months of age for all children born on or after 1^st^ June 2001 and a catch-up campaign for everyone up to the age of 19 years
March 2003	Hepatitis B vaccination added for infants in specified risk groups at 2, 4, and 11 months of age for all children born on or after 1^st^ January 2003
	*Haemophilus influenza* serotype b vaccine given with the DTwP-IPV vaccine at 2, 3, 4, and 11 months of age for all children born on or after 1^st^ April 2002
January 2005	Whole cell pertussis component of the DTwP-IPV vaccine was replaced by the acellular pertussis component at 2, 3, 4, and 11 months of age for all children born after the 1^st^ February 2004
January 2006	Hepatitis B vaccination added at birth for children of mothers who tested positive for HBsAg
June 2006	Pneumococcal vaccination added at 2, 3, 4, and 11 months of age for children born on or after 1^st^ April 2006
	Introduction of combined vaccine DTaP-HBV-IPV/Hib at 2, 3, 4, and 11 months of age for children in specified risk groups born on or after 1^st^ April 2006
July/August 2006	Transition to combined DTaP-IPV vaccine for children at 4 years of age born from July/August 2002 onwards
January 2008	Hepatitis B vaccination added for children with Down syndrome born on or after 1^st^ January 2008
March 2009	Human papillomavirus (HPV) catch-up campaign for girls born 1993-1996
January 2010	HPV vaccination for 12-year-old girls born on or after 1^st^ January 1997
March 2011	The 7-valent pneumococcal vaccine was replaced by the 10-valent pneumococcal vaccine for children born on or after 1^st^ March 2011
August 2011	Hepatitis B vaccination introduced for all children born on or after 1^st^ August 2011

For many years, the childhood vaccination coverage has been high (92-96%; measured at the age of 2 years for newborns, at the age of 5 years for toddlers and at the age of 10 years for schoolchildren [19]) for all target diseases (Table 1), except for HPV, resulting in an highly effective NIP [1]. Therefore, in this study, we assumed all children to be vaccinated according to the Dutch schedule. In addition to the NIP, children with parents originating from tuberculosis endemic countries are invited for vaccination with Bacillus Calmette-Guérin (BCG) vaccine.

Absolute contraindications for vaccination are a proven serious allergy for one of the components of the vaccine or a proven serious allergic reaction following the administration of the same vaccine. Relative contraindications, in which the administration of the vaccine should be considered, include cases of fever (≥38.5 C) and serious immune disorders (disease, treatment with corticosteroids or cytostatics, radiation), following the administration of blood products or immunoglobulins, and patients with an increased tendency toward clotting or bleeding, pregnancy, or planned anaesthesia. However, these cases are quite rare, i.e., 5–1000 cases per year.

### Analysis

**Figure 1:**
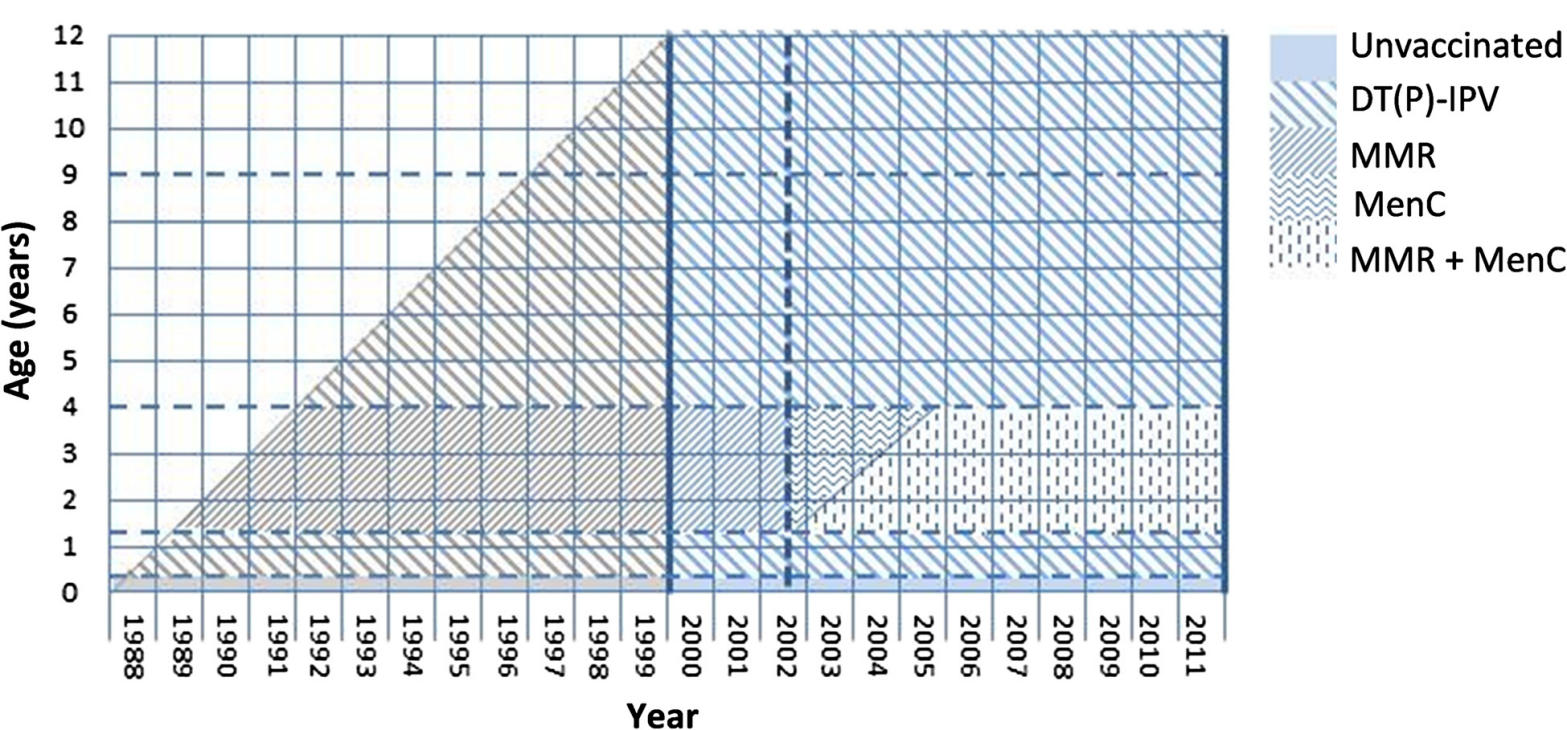
**Lexis diagram of the analysed cohort.** Diagram that represents the type of last vaccination received for the analysed cohort by age and year. The bold vertical lines represent the study period and the dashed vertical line represents the introduction of Meningococcal C vaccination. All changes in the vaccination schedule are presented in Table 2.

The following age groups were created: 0–1 months (i.e., unvaccinated), 2–13 months (i.e., following DT(a)P(−HBV)-IPV(/Hib) (and pneumococcal) vaccination), 14 months to 3 years (i.e., following MMR (and meningococcal C) vaccination (Figure 1)), 4–8 years (i.e., following DT(aP)-IPV booster vaccination) and 9–11 years (i.e., following DT-IPV booster and MMR booster vaccination).

Mortality rates and 95% confidence intervals for all natural causes of death, which was defined as all causes of death other than unknown and external causes of injury and poisoning, were calculated by gender and by age group. Children who died from unknown causes or external causes of injury and poisoning were censored in the analyses. Crude monthly incidence rate ratios (IRR) for girls compared to boys were visualised. IRR, with accompanying 95% confidence intervals, by age group for girls compared with boys were computed using multivariable Poisson regression adjusted for calendar year. To assess whether the IRR differed between age groups, the interaction between age group and gender was tested (degrees of freedom (df) = 4). Furthermore, we tested this interaction for both hypotheses specifically (df = 1), i.e. for 14 months – 3 years of age versus 2–13 months of age and for 4–8 years of age versus 14 months – 3 years of age. In addition, we conducted several sensitivity analyses: 1) including only the indigenous Dutch population, as defined by Statistics Netherlands as children of whom both parents were born in the Netherlands; 2) comparing the periods before and from September 2002 onwards, i.e. before and after the introduction of vaccination against meningococcal C at 14 months of age; 3) including only natural causes of death other than complications of pregnancy, childbirth and the puerperium, conditions originating in the perinatal period and congenital abnormalities; 4) including only infectious and parasitic diseases.

### Ethical statement

The data were made available by Statistics Netherlands. The provided data was fully anonymised. Due to this, approval of an ethics committee was not required and therefore not obtained.

## Results

### Descriptive statistics

The total study population consisted of 6,261,472 individual children, accounting for 30,023,460 person-years. During the study period, 14,038 children (0.22%; mortality rate 4.7/10,000 person years) died. For approximately 9% of the deceased children, an unknown or non-natural cause of death was recorded (Table 3), and these children were censored in the analysis.

**Table 3:** **Characteristics of the study population**

**Characteristic**	**Boys**	**Girls**	**Total**
	**n (%)**	**n (%)**	**N (%)**
*Population 0–11 yr*	*3,188,004*	*3,073,468*	*6,261,472*
Indigenous Dutch	2,292,021 (71.9)	2,189,282 (71.2)	4,481,303 (71.6)
1^st^ generation immigrant	306,934 (9.6)	322,167 (10.5)	629,101 (10.0)
2^nd^ generation immigrant	589,049 (18.5)	562,019 (18.3)	1,151,068 (18.4)
*Deaths*	*7,924*	*6,114*	*14,038*
0-1 months^a^	4,713 (59.9)	3,688 (60.3)	8,401 (59.8)
2-13 months^b^	1,143 (14.4)	870 (14.2)	2,013 (14.3)
14 months – 3 years^c^	902 (11.4)	671 (11.0)	1,573 (11.2)
4-8 years^d^	766 (9.7)	557 (9.1)	1,323 (9.4)
9-11 years^e^	400 (5.0)	328 (5.4)	728 (5.2)
*Primary cause of death*			
Unknown	128 (1.6)	122 (2.0)	250 (1.8)
Infectious and parasitic diseases	203 (2.6)	171 (2.8)	374 (2.7)
Neoplasms	512 (6.5)	393 (6.4)	905 (6.4)
Diseases of the blood and blood forming organs and certain disorders involving the immune mechanism	53 (0.7)	46 (0.8)	99 (0.7)
Endocrine, nutritional and metabolic diseases	196 (2.5)	148 (2.4)	344 (2.5)
Mental and behavioural disorders	23 (0.3)	19 (0.3)	42 (0.3)
Diseases of the nervous system	383 (4.8)	337 (5.5)	720 (5.1)
Diseases of the circulatory system	176 (2.2)	134 (2.2)	310 (2.2)
Diseases of the respiratory system	162 (2.0)	125 (2.0)	287 (2.0)
Diseases of the digestive system and the genitourinary system	99 (1.2)	61 (1.0)	160 (1.1)
Diseases of the skin, subcutaneous tissue, musculoskeletal system and connective tissue	13 (0.2)	17 (0.3)	30 (0.2)
Complications of the pregnancy, childbirth and the puerperium and conditions originating in the perinatal period	2,973 (37.5)	2,192 (35.9)	5,165 (36.8)
Congenital anomalies	1,926 (24.3)	1,630 (26.7)	3,556 (25.3)
Symptoms, signs and ill-defined conditions	426 (5.4)	332 (5.4)	758 (5.4)
External causes of injury and poisoning	651 (8.2)	387 (6.3)	1,038 (7.4)

^a^Most recent vaccination: none.

^b^Most recent vaccinations: DTaP(−HBV)-IPV/Hib and pneumococcal vaccination.

^c^Most recent vaccinations: MMR and meningococcal C vaccination (only after September 2002).

^d^Most recent vaccination: DTaP-IPV booster vaccination.

^e^Most recent vaccinations: DT-IPV booster and MMR booster vaccination.

### Mortality rates

The mortality rate due to natural causes was highest for children 0 to 1 months old. This high mortality rate was mainly caused by the relatively high mortality during the first day after birth, which accounted for 25% of all childhood mortality occurring in the first month of life. In all age groups, the mortality rate was higher among boys than girls (Table 4). Mortality rates declined over the years from 2000 to 2011 (for boys 4.0 in 2000 to 2.8 in 2011; for girls 3.4 in 2000 to 2.1 in 2011).

**Table 4:** **Crude mortality rates due to natural causes in children with respect to gender and age group, from 2000–2011, and adjusted incidence rate ratios (IRR) for girls compared with boys**

**Age group**	**Boys**	**Boys**	**Girls**	**Girls**	**IRR** ^**a**^	**p-value**
	**Number of deaths**	**Mortality rate/10,000 person**	**Number of deaths**	**Mortality rate/10,000 person**	**(95% CI)**	
	**(N = 7145)**	**Years (95% CI)**	**(N = 5605)**	**Years (95% CI)**		
0-1 months	4578	32.1 (31.2-33.1)	3555	26.2 (25.3-27.0)	0.81 (0.78-0.85)	
2-13 months	1035	4.6 (4.3-4.8)	797	3.7 (3.4-3.9)	0.81 (0.74-0.89)	
14 months – 3 years	680	1.4 (1.3-1.5)	551	1.2 (1.1-1.3)	0.85 (0.76-0.95)	0.493^b^
4-8 years	552	0.7 (0.7-0.8)	441	0.6 (0.5-0.7)	0.84 (0.74-0.95)	0.868^c^
9-11 years	300	0.6 (0.5-0.6)	261	0.5 (0.5-0.6)	0.91 (0.77-1.07)	
						0.723^d^

^a^Adjusted for calendar year.

^b^p-value for interaction (df = 1) between age group and gender for 14 months – 3 years of age versus 2–14 months of age.

^c^p-value for interaction (df = 1) between age group and gender for 4–8 years of age versus 14 months – 3 years of age.

^d^p-value for interaction (df = 4) between age group and gender for all age groups.

**Figure 2:**
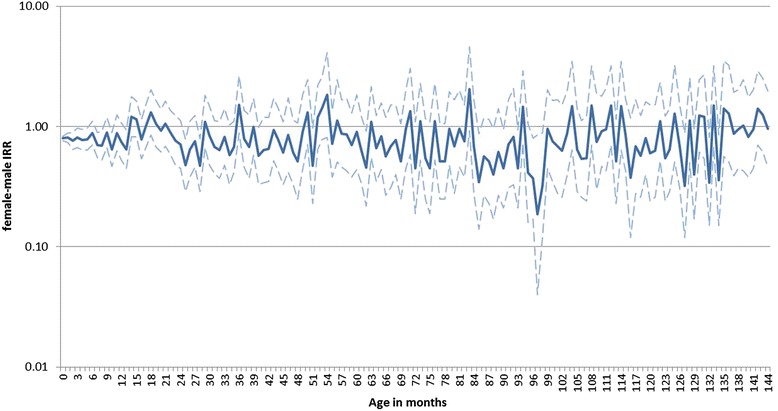
**Female-to-male incidence rate ratios up to 12 years of age.** Plot of crude incidence rate ratios (IRR), including 95% confidence intervals, for girls compared to boys for mortality due to natural causes, from 2000–2011, by month of age up to 12 years.

The plot of female-to-male IRR shows that IRR fluctuated over age in months, however, intervals were overlapping (Figure 2). IRRs fluctuated over the years, especially for children 9–11 years of age (data not shown). The adjusted IRRs for girls compared with boys ranged between 0.81 (95% CI 0.74-0.89) and 0.91 (95% CI 0.77-1.07) in the age groups (Table 4). In particular, the IRR for children 2 to 13 months-old was 0.81 (95% CI 0.74-0.89) and for children 14 months-old to 3 years-old the IRR was 0.85 (95% CI 0.76-0.95). Importantly, no statistically significant differences in IRRs were observed between all age groups (p = 0.723), between children 2–13 months and 14 months - 3 years (p = 0.493) and between children 14 months - 3 years and 4–8 years old (p = 0.868). In sensitivity analyses for indigenous Dutch children only, for periods before and from September 2002 onwards, for natural causes of death other than complications of pregnancy, childbirth, perinatal period and congenital abnormalities, and for infectious and parasitic diseases only, no statistically significant differences in IRR between vaccine-related age groups were found (Table 5).

**Table 5:** **Incidence rate ratios (IRR) for girls compared with boys for indigenous Dutch children only, for the periods before and from September 2002 onwards, and for natural causes of death other than complications of pregnancy, childbirth, perinatal period and congenital abnormalities**

**Age group**	**IRR** ^**a**^	**p-value**	**IRR** ^**a**^	**p-value**	**IRR** ^**a**^	**p-value**	**IRR** ^**a**^	**p-value**	**IRR** ^**a**^	**p-value**
	**(95% CI) for natural causes for indigenous Dutch children only** **(N**_**deaths**_ **= 9,328)**		**(95% CI) for natural causes before September 2002** **(N**_**deaths**_ **= 3,529)**		**(95% CI) for natural causes from September 2002 onwards** **(N**_**deaths**_ **= 9,221)**		**(95% CI) for natural causes other than complications of pregnancy, childbirth, perinatal period and congenital abnormalities** **(N**_**deaths**_ **= 4,029)**		**(95% CI) for infectious and parasitic diseases** **(N**_**deaths**_ **= 374)**	
0-1 months	0.80 (0.76-0.84)		0.81 (0.74-0.88)		0.82 (0.78-0.86)		0.97 (0.82-1.15)		1.13 (0.67-1.90)	
2-13 months	0.78 (0.70-0.87)		0.86 (0.73-1.02)		0.79 (0.71-0.88)		0.74 (0.65-0.84)		0.79 (0.55-1.13)	
14 months – 3 years	0.79 (0.69-0.90)	0.882^b^	0.81 (0.66-0.99)	0.637^b^	0.87 (0.76-0.99)	0.266^b^	0.82 (0.73-0.93)	0.789^b^	0.90 (0.63-1.27)	0.621^b^
4-8 years	0.83 (0.72-0.96)	0.637^c^	0.75 (0.59-0.96)	0.652^c^	0.87 (0.75-1.01)	0.973^c^	0.85 (0.74-0.97)	0.217^c^	0.95 (0.52-1.75)	0.863^c^
9-11 years	0.92 (0.75-1.11)		0.72 (0.52-0.98)		1.00 (0.82-1.21)		0.89 (0.75-1.06)		0.71 (0.33-1.54)	
		0.673^d^		0.835^d^		0.248^d^		0.112^d^		0.817^d^

^a^Adjusted for calendar year.

^b^p-value for interaction (df = 1) between age group and gender for 14 months – 3 years of age versus 2–14 months of age.

^c^p-value for interaction (df = 1) between age group and gender for 4–8 years of age versus 14 months – 3 years of age.

^d^p-value for interaction (df = 4) between age group and gender for all age groups.

## Discussion

Childhood mortality in the Netherlands was very low and declined further over the period from 2000–2011. Mortality from natural causes between birth and 12 years of age was consistently lower among girls than among boys. We observed no significant difference in the female-to-male IRR in DTP-IPV- and MMR ± MenC-eligible children with respect to the last scheduled vaccination.

A major strength of this study is that near complete mortality data were obtained for a long time period from the national registry for causes of death and included the entire Dutch population. However, this study also has some limitations. We could not verify the individual vaccination status of the children and assumed that vaccinations were given as scheduled. A recent study observed that 82% of the vaccinated population received the first DTaP-IPV within 9 weeks of age (<70 days) [20]. The median age at vaccination was 62 days (5th to 95th percentile 51–81 days). Vaccination coverage (personal communication A. van Lier) and delay in vaccination [20] in the Netherlands does not differ by sex. However, delay in vaccination may have resulted in an underestimation of the effect of the last received vaccination on mortality.

Furthermore, changes in the vaccination schedule were made during the study period. *Haemophilus influenza*e type b and hepatitis b components were added to the DTP-IPV vaccine in 2003 and 2011, respectively and in 2006 pneumococcal vaccination was introduced simultaneously. In September 2002 vaccination against meningococcal C was introduced for children 14 months of age together with a catch-up campaign for children up to 19 years of age. This could possibly have interfered with the potential effect of MMR vaccination and therefore sensitivity analysis was done to compare the periods before and from September 2002 onwards. This analysis did not change our conclusions. Additionally, children with parents originating from tuberculosis endemic countries are offered vaccination with the BCG vaccine in addition to vaccinations through the NIP. BCG vaccination could potentially interfere with the results of the study. To exclude this, we conducted a sensitivity analysis including indigenous Dutch children only, which resulted in similar findings. Also, we conducted sensitivity analysis for natural causes of death except for pregnancy, childbirth and perinatal complications and congenital abnormalities to exclude causes of death which were not related to immune system complications. This analysis did also not change our conclusion. Finally, analysis for children who died due to infectious or parasitic diseases only also has not led to other results, although numbers were low.

We did not observe higher mortality among girls than boys following DTP-IPV vaccination in conjunction with a significant declining female-to-male IRR after measles vaccination as reported previously in countries with high childhood mortality [6,10,13-15,17]. In our study, female-to-male IRRs were less than 1 for all age groups and did not differ between age groups. A possible explanation for the different findings could be the major difference in populations and in setting between our study and previous studies. Our study was conducted in a high-income country with low mortality among children, whereas the other studies were conducted in low-income settings with high childhood mortality. In the latter setting, healthcare is less accessible, infectious disease burden from non-vaccine preventable diseases can be significantly higher, and childhood nutrition might be less balanced. These conditions may exhibit a differential impact on girls versus boys or on vaccinated versus unvaccinated children, as reasons for non-vaccination are unlikely to be random, leading to a potential selection bias [21]. Furthermore, it might be more difficult to collect complete validated data on childhood mortality as well as detailed and reliable information on vaccination status, leading to survival bias [8,21,22]. In addition, the non-specific effects of vaccination are less likely to influence overall mortality in high-income settings because of the low prevalence of infectious diseases and the small number of children who die due to infectious diseases. It would be more sensitive to assess these non-specific effects on morbidity. In Denmark, a high-income county, receipt of the MMR vaccine versus the DTaP-IPV-Hib vaccine as the most recent vaccine was found to be associated with a lower rate of hospital admissions due to infections [23]. However, no difference was observed between boys and girls, which indicate that non-specific effects are not necessarily sex-differential.

## Conclusions

In conclusion, we did not find indications for sex-differential non-specific effects on mortality related to the last scheduled vaccination in DTP-IPV- and MMR ± MenC-eligible children in the Netherlands. These study results are important for public health policies. Ongoing evaluation of specific and potential non-specific effects of vaccinations on mortality and morbidity in high-income countries is needed to guide policy, to provide accurate information of the benefits and risks of vaccination and to maintain public trust in public health interventions, such as vaccination.

## Abbreviations

BCG: Bacillus Calmette-Guérin
CI: Confidence interval
Df: Degrees of freedom
DTP: Diphtheria, tetanus, pertussis
DTaP: Diphtheria, tetanus, acellular pertussis
GBA: Municipal Personal Records Database
HBV: Hepatitis B virus
Hib: Haemophilus influenza type b
HPV: Human papillomavirus
IPV: Inactivated polio vaccine
IRRs: Incidence rate ratios
MMR: Mumps, measles, rubella
MenC: Meningococcal serotype C
NIP: National Immunisation Programme
Pneumo: Pneumococcal
